# Modeled nitrate levels in well water supplies and prevalence of abnormal thyroid conditions among the Old Order Amish in Pennsylvania

**DOI:** 10.1186/1476-069X-11-6

**Published:** 2012-02-17

**Authors:** Briseis Aschebrook-Kilfoy, Sonya L Heltshe, John R Nuckols, Mona M Sabra, Alan R Shuldiner, Braxton D Mitchell, Matt Airola, Theodore R Holford, Yawei Zhang, Mary H Ward

**Affiliations:** 1 Occupational and Environmental Epidemiology Branch, Division of Cancer Epidemiology and Genetics, National Cancer Institute, National Institutes of Health, Department of Health and Human Services, Rockville, MD, USA; 2 Yale School of Public Health, Yale University, New Haven, Connecticut, USA; 3 Biostatistics Branch, Division of Cancer Epidemiology and Genetics, National Cancer Institute, National Institutes of Health, Department of Health and Human Services, Rockville, MD, USA; 4 Department of Environmental and Radiological Health Sciences, Colorado State University, Fort Collins, CO, USA; 5 Endocrine Service, Department of Medicine, Memorial Sloan-Kettering Cancer Center, New York, NY, USA; 6 Division of Endocrinology, Diabetes and Nutrition, Department of Medicine, University of Maryland School of Medicine, Baltimore, MD, USA; 7 Geriatrics Research and Education Clinical Center, Veterans Administration Medical Center, Baltimore, MD, USA; 8 Westat, Rockville, MD, USA; 9 Department of Health Studies, University of Chicago, Chicago, IL, USA

**Keywords:** Nitrate, Thyroid Conditions, TSH, Old Order Amish, Water pollution, Drinking water

## Abstract

**Background:**

Nitrate is a widespread contaminant of drinking water supplies, especially in agricultural areas. Nitrate intake from drinking water and dietary sources can interfere with the uptake of iodide by the thyroid, thus potentially impacting thyroid function.

**Methods:**

We assessed the relation of estimated nitrate levels in well water supplies with thyroid health in a cohort of 2,543 Old Order Amish residing in Lancaster, Chester, and Lebanon counties in Pennsylvania for whom thyroid stimulating hormone (TSH) levels were measured during 1995-2008. Nitrate measurement data (1976-2006) for 3,613 wells in the study area were obtained from the U.S. Geological Survey and we used these data to estimate concentrations at study participants' residences using a standard linear mixed effects model that included hydrogeological covariates and kriging of the wells' residuals. Nitrate levels estimated by the model ranged from 0.35 mg/L to 16.4 mg/L N-NO_3_^-^, with a median value of 6.5 mg/L, which was used as the cutpoint to define high and low nitrate exposure. In a validation analysis of the model, we calculated that the sensitivity of the model was 67% and the specificity was 93%. TSH levels were used to define the following outcomes: clinical hyperthyroidism (n = 10), clinical hypothyroidism (n = 56), subclinical hyperthyroidism (n = 25), and subclinical hypothyroidism (n = 228).

**Results:**

In women, high nitrate exposure was significantly associated with subclinical hypothyroidism (OR = 1.60; 95% CI: 1.11-2.32). Nitrate was not associated with subclinical thyroid disease in men or with clinical thyroid disease in men or women.

**Conclusions:**

Although these data do not provide strong support for an association between nitrate in drinking water and thyroid health, our results do suggest that further exploration of this hypothesis is warranted using studies that incorporate individual measures of both dietary and drinking water nitrate intake.

## Background

Nitrate is a widespread contaminant of drinking water supplies, especially in agricultural areas. The thyroid can concentrate univalent anions such as nitrate (NO_3_^-^), which subsequently interferes with the uptake of iodide (I^-^) by the thyroid and may cause reduced production of thyroid hormones [1-4]. The result of the reduced thyroid hormone production is a compensatory increase in thyroid stimulating hormone (TSH), a sensitive indicator of thyroid function. High and low TSH levels reflect hypo- and hyperfunction of the thyroid gland, respectively. Chronic stimulation of the thyroid gland by excessive TSH has been shown in animals to induce the development of hypertrophy and thyroid disease, as well as hyperplasia, followed by adenoma and carcinoma [5]. At least two epidemiological studies have shown high nitrate intake to be associated with thyroid dysfunction, including hypertrophy and changes in TSH levels [6,7]; however, the impact of nitrate intake on specific thyroid conditions, including hyperthyroidism and hypothyroidism is not clear.

Elevated concentrations of nitrate in groundwater originate from a number of sources, including leaking septic tanks, animal waste, and overuse of nitrogen fertilizers [7]. Nitrate is very soluble and it readily migrates to groundwater. Nitrate contamination of groundwater is an exposure of interest as groundwater serves as the primary drinking water supply for over 90% of the rural population and 50% of the total population of North America [8]. Although the U.S. Environmental Protection Agency (EPA) maximum contaminant level (MCL) for nitrate as nitrogen (nitrate-N) is 10 mg/L in public water sources [9], the levels in private wells are not regulated and the task of monitoring is left to residential owners, presenting opportunities for high levels of human exposure. The U.S. Geological Survey (USGS) estimates that nitrate concentrations exceed the EPA's standard in approximately 15% of agricultural and rural areas, exposing over 2 million people in the United States [10]. The MCL for nitrate in drinking water was established to protect against methemoglobinemia, or "blue baby syndrome," to which infants are especially susceptible. However, this health guideline has not been thoroughly evaluated for other health outcomes such as thyroid disease and cancer.

The Old Order Amish community is a population characterized by a homogeneous lifestyle, including intensive farming practices and low mobility, and has been relatively unchanged across generations [11]. In areas where many large dairy and poultry farms are concentrated, the land area for disposal of animal wastes is limited. This situation often results in overloading the available land with manure, with considerable nitrogen ending up in groundwater or surface water [8,12]. Lancaster County in southeastern Pennsylvania is an example of such an area where extensive dairy enterprises with high stocking rates prevail. High levels of nitrate in the groundwater [8] suggest that the Amish are a potentially highly exposed population. Given the biological effects of nitrate intake on the thyroid, investigation of whether the Amish in this area exhibit an increased prevalence of thyroid dysfunction and thyroid disease is of interest.

The aim of this study is to assess whether nitrate concentrations in well water are associated with levels of TSH and thyroid disease. Our goal was to use survey data on nitrate levels in well-water obtained from the USGS to conduct a cross-sectional analysis of the association between nitrate exposure and thyroid health. This study builds upon several ongoing studies of diabetes, obesity, osteoporosis, hypertension, and cardiovascular disease in the Amish, initiated in 1993 at the University of Maryland [13-16].

## Methods

### Study population

Subjects included in this analysis were 3,017 Old Order Amish aged 18 years and older from Lancaster, Chester, and Lebanon Counties, Pennsylvania, for whom thyroid health was assessed through measurement of thyroid stimulating hormone (TSH) levels in their prior participation in one or more studies of health by investigators at the University of Maryland, Baltimore [13-16]. We excluded participants whose residences were located outside of Lancaster, Chester, or Lebanon counties (n = 328) due to sparse nitrate measurement data, and persons who reported use of thyroid medication (n = 145) leaving a total of 2,543 persons (1,336 females and 1,207 males) in the final analysis. Nearly all of the enrolled individuals are descendants of a small number of Amish who settled in Lancaster County, Pennsylvania, in the mid-eighteenth century [13,17,18]. This study was approved by the Institutional Review Boards of the University of Maryland and the National Cancer Institute.

All subjects included in this analysis received a standardized examination at the Amish Research Clinic in Strasburg, Pennsylvania or in the participant's home during the time period 1995-2008. As part of this examination, a fasting blood sample was collected from which TSH levels were measured with the Siemens TSH assay (Immulite 2000; Deerfield, IL) according to the manufacturer's instructions. The method is a solid-phase, chemiluminescent, competitive analog immunoassay and has analytical sensitivity of 0.004 μIU/ml and upper limit of 75 μIU/ml of TSH.

**Figure 1:**
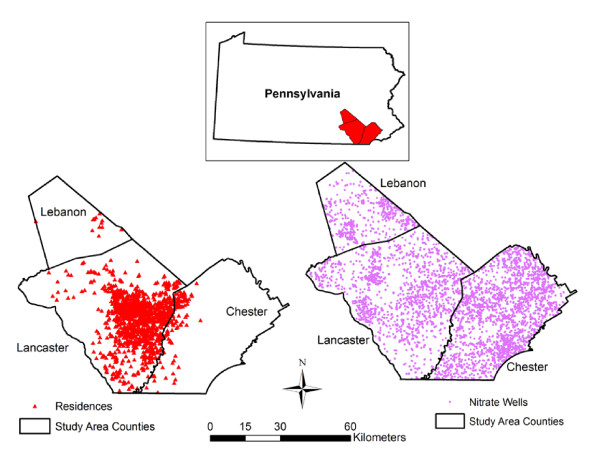
**Location of participant residences and wells with nitrate measures in study area**.

Residential street addresses were geocoded using the TeleAtlas (Lebanon, NH) MatchMaker SDK Professional version 8.3 (October 2006), a spatial database of roads, and a modified version of a Microsoft Visual Basic version 6.0 program issued by TeleAtlas to match input addresses to the spatial database. We assigned residence location using an offset of 25 ft from the street centerline. Addresses that were not successfully geocoded were checked for errors using interactive geocoding techniques. Where only a street intersection was available for the residential location (1.0% of residences), we assigned the geographic location of the residence to the middle of the intersection. Where only a zip code was available for the residential location (3.0% of residences), we assigned the geographic location of the residence to the centroid of the zip code. The geocoded location of the residences and the geographic boundary of our study area is shown in Figure 1.

### Historical assessment of nitrate levels

A survey of nitrate levels in well water in Lancaster, Chester, and Lebanon counties was carried out from 1976-2006 by the USGS. The USGS collected data from active monitoring wells in the county and from a well-owner monitoring program conducted by the State Department of Natural Resources in collaboration with Pennsylvania State University. Water samples (50-100 uL) from all programs were measured for nitrate using ion chromatography with a detection limit of 0.002 mg/L as nitrate-N [19].

A total of 3,613 unique wells were measured in our study area during the survey period. The measurements were not from wells chosen at random but included monitoring data reported by the USGS and samples from individual well owners. A total of 3,057 wells had 1 measurement; 198 wells had 2 measurements; 113 wells had 3 measurements; and 245 wells had more than 3 measurements. Figure 1 shows the geographic distribution of the wells in our study area in relation to the location of the 2,543 participant residences The median distance between a residence and the closest measured well was 576.0 m (interquartile range: 308.0-897.2 m). The median nitrate concentration by season ranged from 2.0 mg/L as nitrate-nitrogen (hereafter mg/L) for summer months (interquartile range: 0-7.5 mg/L) to 2.7 mg/L in spring months (interquartile range: 0-8.8 mg/L). For wells with multiple measures, the median difference between the maximum and minimum value was 1.2 mg/l (IQR: 0.3-0.9). The mean of the measurements was used for wells with multiple measurements when we did the exposure modeling (see below).

### Prediction of nitrate levels in well water of participants' residences

**Figure 2:**
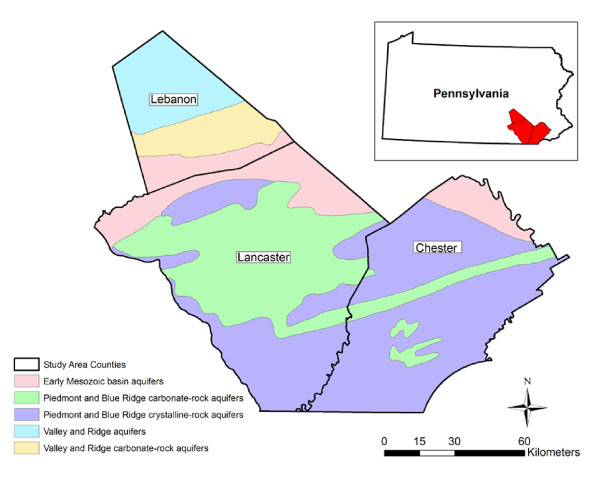
**Principle aquifers in the three study area counties in southeastern Pennsylvania**. Data from Principal Aquifers of the 48 Conterminous United States, Hawaii, Puerto Rico, and the U.S. Virgin Islands: U.S. Geological Survey. Madison, WI; 2003

**Figure 3:**
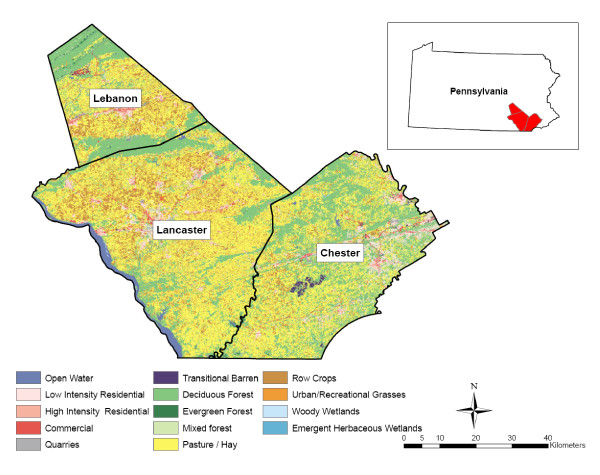
**Land use in 1992 in the three study area counties in southeastern Pennsylvania**. Data from Principal Aquifers of the 48 Conterminous United States, Hawaii, Puerto Rico, and the U.S. Virgin Islands: U.S. Geological Survey. Madison, WI; 2003

We assumed the drinking water supply for participants to be a well located at their reported residence. To estimate nitrate levels at this location, we first determined whether nitrate concentrations in the USGS wells varied across the types of aquifers in the study area (Table 1). Maps of the primary aquifers were obtained from the USGS (created from 30 m pixel satellite imagery) [19]. There are five principal aquifers in the study area (Figure 2). The differentiation of aquifer type is important because the transport of contaminants in groundwater is generally confined to within these hydrogeologic boundaries. Using the 1992 USGS National Land Cover Data Set (NLCD) [20], we also evaluated nitrate levels in the USGS wells across thirteen types of land use (pasture, deciduous forest, row crops, low intensity residential, mixed forest, commercial or industrial, evergreen forest, high intensity residential, water, quarries/gravel pits, transitional, urban grasses, and woody wetlands) (Table 1 and Figure 3). We found limited temporal variation by season and by decade within each of the five aquifer types over the well measurement period, as well as across the land use classifications used in our analysis (Figure 3).

**Table 1:** Distribution of nitrate concentration in US Geological Survey wells by aquifer type and categories of land use in Lancaster, Lebanon, and Chester Counties, from 1976-2006

Well Location			Nitrate mg/L (*NO3-N*)			
**Aquifer**	**N**	**Median**	**Mean**	**Std. Dev**.	**Minimum**	**Maximum**

Piedmont and Blue Ridge crystaline-rock	1676	3.00	4.35	4.88	< .002	52.00

Piedmont and Blue Ridge carbonate rock	1093	6.03	7.88	7.43	0.020	95.50

Early mesozoic basin	469	3.77	4.96	5.28	< .002	45.00

Valley and ridge carbonate rock	241	11.00	13.60	13.39	< .002	90.00

Valley and ridge	134	1.02	2.12	2.86	0.010	16.64

**Land use 1992**	N	Median	Mean	Std. Dev.	Minimum	Maximum

Pasture	1756	5.00	6.89	7.37	< .002	90.00

Deciduous forest	674	2.15	3.60	4.46	0.003	45.00

Row crop	399	6.91	9.24	10.79	0.020	95.50

Low intensity residential	289	3.68	4.49	4.91	0.003	52.00

Mixed forest	173	2.50	3.81	3.84	< .002	20.80

Commercial or industrial	130	4.94	5.76	4.66	0.020	22.10

Evergreen forest	121	3.63	4.85	4.37	0.003	21.50

High intensity residential	21	5.95	5.09	2.47	0.690	9.23

Water	17	3.72	4.61	5.39	0.020	22.00

Quarry mine gravel pit	15	0.80	2.02	3.69	0.150	15.00

Transitional	11	1.78	2.34	1.81	0.190	6.13

Urban grasses	4	5.39	4.86	2.68	1.420	7.26

Woody wetland	3	2.48	6.22	8.45	0.280	15.90

**Overall**	3613	**4.12**	**6.03**	**7.1**	**< .002**	**95.5**

Spatial classification is based on the National Land Cover Data Set; 1992, USGS [ref 38].

We used a standard linear mixed effects statistical model to develop a predictive model including the variables principal aquifer and land use. Nitrate levels were log normally distributed so we modeled the natural logarithm of the concentration. Spatial correlation existed in the nitrate measurements even after covariate adjustment [21], so we performed kriging on the wells' residuals from the predictive nitrate model. If a well had more than one measurement, the mean of the measurements and its residual was used in the modeling. We assumed that the residuals in the model have a single, normally distributed mean structure centered at zero, allowing for universal kriging across the study area. The kriging procedure predicts a 'residual' for each study participant based on a weighted average of the 20 neighboring wells' residuals (within the respective aquifer and land use category). For comparison, we also applied the kriging procedure based on the weighted average of the five neighboring wells' residuals. For example, if for a particular region of our study area, the regression model tends to underestimate the true observed log nitrate values (positive residuals) then individuals in this region will be given a representative positive residual prediction that is added to the log nitrate estimate based on the individuals' covariates and the regression parameters. The antilog gives an unbiased predictor of median nitrate value resulting in estimates that are more robust to outlier observations than a mean estimator. Nitrate levels estimated by the model ranged from 0.35 mg/L to 16.4 mg/L, with a median of 6.5 mg/L and a mean of 6.6 mg/L (sd = 2.9 mg/L). The predicted nitrate level mean was similar to the mean of the measured values used for modeling (6.8 mg/L; sd = 8.3 mg/L) although the standard deviation was smaller.

### Model validation

The validity of the predictive model was assessed for 77 validation wells by comparing the predicted nitrate concentration to the observed nitrate concentrations. The validation wells were randomly selected from 482 wells with USGS measurements from 1992-1993. The limited date range was chosen to be consistent with the time frame of the NLCD land use database used in our analyses. We evaluated model sensitivity, specificity, and percent agreement using the median of the predicted nitrate level (6.5 mg/L as nitrate-N) as a cutpoint for high and low exposure categories. The sensitivity of the model was 67% and the specificity was 93%. The Spearman's rank correlation between the continuous predicted and measured concentrations was 0.73. Cross tabulation of predicted and observed nitrate concentrations by quartiles of the measured nitrate concentrations demonstrated a percent agreement of 56% (Table 2).

**Table 2:** Comparison of quartiles of the predicted nitrate concentration by quartiles of the measured nitrate concentrations, 77 wells used for the model validation

Quartiles of the predicted nitrate concenytration					
**Quartiles of measured nitrate concentration**	**Q1**	**Q2**	**Q3**	**Q4**	**Total**

Q1	13	1	3	0	17

Q2	3	11	6	0	20

Q3	1	6	6	6	19

Q4	1	2	5	13	21

total	18	20	20	19	77

Percent agreement = 56%

### Data analysis

We used generalized linear regression to assess the association between estimated nitrate levels in well water and continuous TSH measures. TSH levels were also used to define disease status based on clinical guidelines [21]. A "normal" range for TSH was defined as 0.4-4 mIU/ml. A TSH level of > 4 mIU/ml-10 mIU/ml was defined as subclinical hypothyroidism (n = 228) and more than 10 mIU/ml was defined as clinical hypothyroidism (n = 56). A TSH value of 0.1 mIU/ml to 0.4 mIU/ml was defined as subclinical hyperthyroidism (n = 25) and less than 0.1 mIU/ml was defined as clinical hyperthyroidism (n = 10). All of the disease definitions are based on the assumption that TSH was marking primary disease in the thyroid since other causes of TSH abnormalities, e.g., primary pituitary disease, thyroid hormone resistance, are very uncommon by comparison [22].

Estimated nitrate levels in participants' drinking water were categorized into quartiles and by the median of the predicted well nitrate level (6.5 mg/L). We evaluated the association of the nitrate levels with each thyroid disease group using unconditional logistic regression to compute the odds ratio (OR) and 95% confidence intervals. All models were adjusted for potential confounding factors including age (continuous) and BMI ((normal (< 25 kg/m^2^), overweight (25-30 kg/m^2^), and obese (> 30 kg/m^2^)). We conducted analyses stratified by gender as well as for men and women combined. Tests of linear trend were performed by modeling the continuous nitrate estimates. A p-value < 0.05 was considered significant and all data analyses were conducted using SAS version 9.1.

We conducted two sensitivity analyses. In the first analysis, we excluded participants whose residences were located within boundaries of the U.S. Census Places (USCB 2004) and were therefore possibly connected to public water supplies with nitrate levels below the MCL (16% of study population). In the second analysis, we excluded those whose residence was greater than 1500 m from the nearest well with measurement data (17%) to reduce the probability of measurement error. We recomputed the OR for subclinical hypothyroidism after correcting for exposure misclassification (i.e. by reclassifying false positives and false negatives) using our estimates of sensitivity and specificity and the prevalence of exposure (50%).

## Results

The mean TSH level was 2.92 mIU/ml (3.05 mIU/ml for women and 2.77 mIU/ml for men). Based on the TSH measures, the prevalence of clinical hyperthyroidism was 0.4% and the prevalence of subclinical hyperthyroidism was 1.0%. The prevalence of clinical hypothyroidism was 2.2% and the prevalence of subclinical hypothyroidism was 9.0%.

The mean age of participants was 50 years (range: 18-98). The mean BMI was 26.6 kg/m^2 ^for men and 27.7 kg/m^2 ^for women (Table 3). The average BMI of males with clinical hyperthyroidism was lower than that of those in the general study population but females with clinical hyperthyroidism had a slightly higher average BMI than the general study population. The average age of persons with thyroid disease was higher in all categories compared to the group with normal TSH levels. Although smoking data was not available for the entire study population, among those for whom these data were collected, less than 1% of women (4 of 657) and 43% of males (310 of 725) reported ever smoking tobacco.

**Table 3:** Characteristics of the study population by thyroid disease status

	Normal	Subclinical Hyperthyroidism	Clinical Hyperthyroidism	Subclinical Hyperthyroidism	Clinical Hyperthyroidism	Total
**Overall (N, %)**	2224 (87.5%)	25 (1.0%)	10 0.4%)	228 (9.0%)	56 (2.2%)	2,543

**Male (%)**	48.6	32.0	30	40.8	41.1	47.5

**Age in years (mean)**	48.9	56.9	56.2	59.2	56.7	50.1

**BMI Males kg/m2 (mean)**	26.6	27.2	22.3	26.7	27.5	26.6

**BMI Females kg/m2 (mean)**	27.5	27.5	27.1	28.5	29.2	29.2

Ever Smoker (%)*	22.3	16.7	33.3	26.5	26.3	22.7

Includes persons who reside in Lancaster, Chester, or Lebanon Counties and excludes persons younger than 18 or who report use of thyroid medications.

*smoking status is based on 45.8% of the study population

Adjusting for age and BMI, and modeling TSH concentration as the outcome, we observed no significant relationship with nitrate concentration. The *B *coefficient for men and women combined was -0.12 (p-value = 0.14), -0.13 for men (p-value = 0.11), and -0.12 for women (p-value = 0.40). Modeling the dichotomized high/low nitrate predictor, the *B *coefficient for men and women combined was -0.64 (p-value = 0.19), -0.57 for men (p-value = 0.22), and -0.70 for women (p-value = 0.40).

Neither clinical or subclinical hyperthyroidism were associated with nitrate concentrations (Table 4), although the number of cases was low (n = 10 cases of clinical hyperthyroidism and n = 25 cases of subclinical hyperthyroidism).

**Table 4:** Odds ratios (ORs) and 95% confidence intervals (CIs) for the prevalence of hyperthyroidism associated with estimated nitrate levels in residential wells

*Overall*			*Men*		*Women*	
**mg/L nitrate-nitrogen**	**Cases**	**OR 95%CI**	**Cases**	**OR 95%CI**	**Cases**	**OR 95%CI**

**Clinical Hyperthyroidism**						

Low nitrate (< 6.5)	5	1.0	1	1.0	4	1.0

High nitrate (= > 6.5)	5	0.95 (0.27-3.28)	2	1.85(0.17-20.7)	3	0.70 (0.16-3.15)

**Subclinical Hyperthyroidism**						

Low nitrate (< 6.5)	13	1.0	5	1.0	8	1.0

High nitrate (= > 6.5)	12	0.86(0.39-1.91)	3	0.57 (0.13-2.38)	9	1.07 (0.41-2.79)

**Subclinical Hyperthyroidism**						

Q1 [0.34-4.46]	6	1.0	1	1.0	5	1.0

Q2 [4.47-6.53]	7	1.14 (0.38-3.42)	4	4.01(0.45-36.2)	3	0.53 (0.14-2.47)

Q3 [6.54-8.55]	8	1.29 (0.45-3.76)	2	1.98 (0.18-22.0)	6	1.17 (0.35-3.90)

Q4 [8.56-16.4]	4	0.62 (0.18-2.23)	1	0.99 (0.06-15.9)	3	0.56 (0.13-2.38)

p-trend		0.36		0.25		0.65

Models adjusted for age and BMI; model with men and women combined is also adjusted for gender

The results for hypothyroidism are presented in Table 5. Overall, there was a borderline significant positive association between subclinical hypothyroidism and high nitrate exposure (age- and BMI-adjusted OR = 1.32; 95% CI: 1.0-1.68), with further analyses revealing the association to be present in women (OR = 1.60; 95% CI: 1.11-2.32), but not in men (OR = 0.98; 95% CI: 0.63-1.52). However, the association among women did not increase monotonically with increasing quartiles of estimated nitrate concentrations in their water supply exposure. The interaction for gender and nitrate was not significant (p-interaction = 0.32). No significant associations were observed for clinical hypothyroidism. The results were consistent when stratified by age and BMI.

**Table 5:** Odds ratios (ORs) and 95% confidence intervals (CIs) for the prevalence of hypothyroidism associated with nitrate levels in residential wells

*Overall*			*Men*		*Women*	
mg/L nitrate-nitrogen	**Cases**	**OR 95%CI**	**Cases**	**OR 95%CI**	**Cases**	**OR 95%CI**

**Clinical Hypothyroidism**						

Low nitrate (< 6.5)	29	1.0	11	1.0	18	1.0

High nitrate (= > 6.5)	27	0.89 (0.52-1.52)	12	0.98 (0.43-2.25)	15	0.82 (0.41-1.66)

**Subclinical Hypothyroidism**						

Low nitrate (< 6.5)	96	1.0	44	1.0	52	1.0

High nitrate (= > 6.5)	132	1.32 (1.0-1.75)	49	0.98 (0.63-1.52)	83	**1.60 (1.11-2.32)**

**Subclinical Hypothyroidism**						

Q1 [0.34-4.46]	48	1.0	20	1.0	28	1.0

Q2 [4.47-6.53]	49	0.99 (0.65-1.51)	24	1.06 (0.56-2.00)	25	0.93 (0.52-1.64)

Q3 [6.54-8.55]	69	1.45 (0.97-2.15)	23	0.97 (0.51-1.83)	46	**1.84 (1.11-3.06)**

Q4 [8.56-16.4]	62	1.23 (0.82-1.84)	26	1.12 (0.60-2.09)	36	1.28 (0.75-2.16)

p-trend		0.57		0.91		0.45

Models adjusted for age and BMI; model with men and women combined is also adjusted for gender; nitrate concentrations in mg/L

The results were unchanged in a sensitivity analysis that excluded participants whose residences were possibly connected to public water supplies (data not shown). The exclusion of persons who reside more than 1500 m from the nearest well also did not result in a material change in our results (data not shown), although it did decrease the odds ratio for high nitrate intake and subclinical hypothyroidism in women from 1.60-1.52. We also estimated the OR for subclinical hypothyroidism among women in the absence of exposure misclassification as 2.1 (versus 1.6 observed).

## Discussion

Our results provide limited support for an association between nitrate levels in private wells and subclinical hypothyroidism among women but not men. With estimated exposure to nitrate in drinking water at or above 6.5 mg/L, we observed a significantly increased prevalence of subclinical hypothyroidism in women, although there was not a monotonic increase with increasing quartiles of nitrate. These findings of an increased prevalence of hypothyroidism among women are consistent with our hypothesis, namely that the competitive inhibition of iodide uptake associated with increased nitrate exposure would result in decreased systemic active thyroid hormone (as indicated by increased TSH levels). We did not observe an association for clinical hypothyroidism, but the number of cases in this group was much lower.

The mean TSH level in our study population was 3.05 μIU/ml in women and 2.77 μIU/ml in men. These levels are higher than TSH levels in the general US population surveyed by the National Health and Nutrition Examination Survey (NHANES) from 1988-1994 [23], in which the means among women and men were 1.49 μIU/ml and 1.46 μIU/ml, respectively. The prevalence of hypothyroidism and hyperthyroidism in the US population is estimated to be 4.6% (0.3% clinical and 4.3% subclinical) and 1.3% (0.5% clinical and 0.8% subclinical), respectively [23], compared with 11.2% and 1.4% in our study population. However, as the risk of thyroid disease increases with age, the higher prevalence in the Amish could be partially due to the older age distribution in this study (mean = 50.1 years) population compared to the age distribution of the NHANES study population (mean = 45.0 years). When hypothyroidism is compared by sex in this study population and the NHANES population, the prevalence is 1.5-times more common in Amish women than men whereas it is 2-8 times more common in women than men in the US population [23].

In previous epidemiological studies, investigators have identified a relationship between nitrate contamination of water supplies and thyroid dysfunction and thyroid disease. In a cross-sectional study of school children living in areas of Slovakia with high and low nitrate exposure via drinking water, children in the high nitrate area had increased thyroid volume and increased frequency of signs of subclinical thyroid disorders (thyroid hypoechogenicity by ultrasound, increased TSH level and positive anti-thyroid peroxidase (TPO)) [6]. The nitrate levels ranged from 11.3 to 58.7 mg/L (as nitrate-nitrogen) in the highly polluted area and were < 0.4 mg/L nitrate-nitrogen in the low nitrate area. Similarly, investigators in the Netherlands conducted a cross-sectional study of women who obtained their drinking water from public supplies and private wells with varying nitrate levels [7]. They observed a dose-dependent increase in the volume of the thyroid associated with increasing nitrate concentrations in drinking water from a combination of public and private supplies, with nitrate levels ranging from 0.004 mg/L to 29.1 mg/L (as nitrate-nitrogen). Women with nitrate levels exceeding 11.1 mg/L as nitrate-nitrogen had a significant increased prevalence of thyroid gland hypertrophy. Our results for women are consistent with the findings in Slovakia and indirectly support the associations observed in the Netherlands. However, the reason for our finding of in women but not men is unclear particularly since men consume more water than women on average [23]. It is possible that women may be more sensitive to exposures that perturb the thyroid as indicated by their higher prevalence of thyroid disease [24].

A previous epidemiologic investigation of the association of nitrate intake from public water supplies and diet with the risk of self-reported hypothyroidism and hyperthyroidism was conducted in a cohort of 21,977 older women in Iowa [25]. The investigators found no association between the prevalence of hypo- or hyperthyroidism and nitrate concentrations in public water supplies; nor was there an association for those who were using private wells. However, intake of nitrate from the diet can be a primary source of exposure when drinking water nitrate levels are below the MCL of 10 mg/L nitrate-N [26-29]. In the Iowa study, increasing intake of nitrate from dietary sources was associated with an increased prevalence of hypothyroidism (OR Q4 = 1.24; 95% CI = 1.10-1.40, P for trend = 0.001) while no association was observed with hyperthyroidism [24]. In addition to consumption of tap water, people living in areas with high nitrate concentration in their water supplies may be exposed through their use of water for cooking, irrigation of crops used as a food source, and through milk products from local farm animals. Nitrate is a natural component of plants and is found at high concentrations in leafy vegetables, such as lettuce and spinach, and some root vegetables, such as beets [25]. The lack of dietary questionnaire data in our study is a limitation since estimates of well-water nitrate were below the MCL of 10 mg/L for 89% of participants [25-27]. The lack of dietary information in general likely resulted in exposure misclassification in our study population.

A strength of this study is the availability of valid measures of TSH using study participant serum samples. Although only one measure was available for each study participant, the use of TSH rather than self-reported thyroid disease is likely to more accurately define thyroid disease. Although factors such as pregnancy and obesity can affect TSH, the levels are a reliable index of the biological activity of thyroid hormones. Anti-TPO was not available, which can also be helpful in the diagnosis of thyroid disease as an autoimmune disease. In addition to measuring TSH and anti-TPO in blood, future studies would be further strengthened by the use of ultrasound technology to determine thyroid volume, which could provide insight into nitrate exposure levels that may cause hypertrophy of the thyroid.

An additional strength of our study was that we validated our exposure metric and characterized the sensitivity and specificity based on the median observed versus predicted nitrate level in wells monitored by the USGS in our study area. Specificity was high (93%) indicating that our model classified those with lower nitrate levels accurately. The lower sensitivity (67%) indicated that the model underestimated nitrate concentrations for those with higher levels. The result of this misclassification, if nondifferential by disease status [28], would be to attenuate ORs as we demonstrated for subclinical thyroid disease.

Our study was limited by a lack of information about the study population's complete residential history. However, we know that the majority of this Amish cohort reside in rural areas, with low relocation rates, and that it is typically the women who relocate to live in the homes or on the same land as their husband's family [29]. Most Amish men would subsequently have a stable residential history and exposure to nitrate contamination of well water over time. It is not clear to what degree a complete residential history would have affected our findings for both men and women. It is possible that the association we observed between subclinical hypothyroidism in women was attenuated due to this source of misclassification. The well measurements were also not randomly selected but represent data collected by USGS and individuals that potentially reside in areas with higher levels of nitrate than those who did not receive monitoring attention from USGS or who were not aware of a problem in their well. We identified a large standard deviation for the wells with repeat measures and were unable to fully explore the reasoning beyond having a small proportion (13%) of repeat samples. Additional data on well depth, other hydrogeological factors, or why multiple samples were taken could provide more insight into this observed variation, but was not available.

Our study is also limited by the fact that we did not have data on actual water supply source to the residence, nor personal water consumption. Because most residences were located outside of areas served by public water utilities, we assumed the drinking water supply for participants was a well located at their residence. We did not have data on tap water consumption, and thus the approximate daily intake, which can be an important variable in determining exposure. Most people in the United States drink about 1.5-2 l of water per day [30]. Similarly, because of the attention given to water contamination in the Lancaster area, it is possible that some study participants obtain their water from sources that have been purified via reverse osmosis or from bottled water. These limitations would clearly affect the exposure estimates and result in misclassification of the exposure.

Future work in this area would be enhanced by the assessment of multiple contaminants present in water sources and the general environment that could be simultaneously affecting thyroid health. Multiple environmental pollutants from industrial as well as agricultural activities may be an important consideration for future investigation. Of particular interest is pesticides as there is increasing evidence of their ability to alter thyroid hormone homeostasis, causing thyroid dysfunction and thyroid disease [31,32]. The varied effects of these chemicals on thyroid function could affect study findings. Determination of these exposures should be a future study design consideration.

Furthermore, the effect of contamination from other univalent anions which interfere with the uptake of iodide by the thyroid should be considered in future investigation into the effects of nitrate in drinking water. For example, perchlorate, the oxidizer for solid rocket fuel and a component of some fertilizers, is found in both food and water [33], and interferes with iodide uptake much like nitrate. Similarly, thiocyanate, another univalent anion that causes thyroid dysfunction, is a metabolite from tobacco smoke and is found in certain foods [34-37].

## Conclusions

The present study provides limited evidence that nitrate in residential well water is associated with subclinical hypothyroidism in women but not men. Future studies that include validated biomarkers, as well as individual level nitrate exposure estimates of dietary and drinking water intakes, and an assessment of co-contaminants, are needed to provide information about the relevance of nitrate intake and thyroid disease.

## List of Abbreviations

TSH: Thyroid stimulating hormone; NO^3-^: Nitrate; EPA: U.S. Environmental Protection Agency; MCL: Maximum contaminant level; nitrate-N: Nitrate as nitrogen; USGS: United States Geological Survey; NLCD: National Land Cover Data Set; NHANES: National Health and Nutrition Examination Survey

## Competing interests

The authors declare that they have no competing interests.

## Authors' contributions

BA-K: participated in the conceptualization of the study and the exposure assessment design; conducted data analysis and drafted the manuscript; SH: conducted the nitrate modeling work and gave important intellectual input for the data analysis, and helped draft the manuscript; JN: conceptualized the exposure assessment approach, advised in the creation of the hydrogeological covariates and exposure modeling, and provided important intellectual content; MS: advised on the medical interpretation of the findings, and provided important intellectual content; AS: As the PI of the cohort study, AS designed the study, oversaw the recruitment of subjects and conducted much of the primary data gathering; BM: As a co-PI, BM participated in the design of the study and ongoing study efforts, the interpretation of the results for the nitrate investigation, and drafting of the manuscript; MA: obtained the hydrogelogical dataset, GIS analysis, reviewed the manuscript, and revised it critically for important intellectual content; TH: helped conceptualize the study, provided statistical support and modeling guidance, and provided important intellectual content for the manuscript; YZ: helped conceptualize the study and provided important intellectual content for the manuscript; MW: mentored in the conceptualization of the study, participated in study design, method development and data analysis, helped in the drafting of the manuscript. All authors assisted in the interpretation of results and contributed towards the final version of the manuscript. All authors read and approved the final manuscript.
